# *Enterococcus faecalis* promotes the progression of colorectal cancer via its metabolite: biliverdin

**DOI:** 10.1186/s12967-023-03929-7

**Published:** 2023-02-02

**Authors:** Li Zhang, Jing Liu, Mingxia Deng, Xiangliu Chen, Lushun Jiang, Jiajie Zhang, Lisheng Tao, Wei Yu, Yunqing Qiu

**Affiliations:** 1 State Key Laboratory for Diagnosis and Treatment of Infectious Diseases, National Clinical Research Center for Infectious Diseases, Collaborative Innovation Center for Diagnosis and Treatment of Infectious Diseases, The First Affiliated Hospital, Zhejiang University School of Medicine, Hangzhou, China; 2 Department of Gastric Surgery, Institute of Cancer Research and Basic Medical Sciences of Chinese Academy of Sciences, Cancer Hospital of University of Chinese Academy of Sciences, Zhejiang Cancer Hospital, Hangzhou, China; 3 Center for General Practice Medicine, Department of Infectious Diseases, Zhejiang Provincial People’s Hospital, People’s Hospital of Hangzhou Medical College, Hangzhou, China; 4 Department of Gastroenterology, The People’s Hospital Affiliated to Jiangsu University, Zhenjiang, China

**Keywords:** *Enterococcus faecalis*, Colorectal cancer, Interleukin-8, Vascular endothelial growth factor A, Angiogenesis

## Abstract

**Background:**

*Enterococcus faecalis* (Efa) has been shown to be a “driver bacteria” in the occurrence and development of colorectal cancer (CRC). This study aims to explore the effect of specific metabolites of Efa on CRC.

**Methods:**

The pro-tumor effects of Efa were assessed in colonic epithelial cells. The tumor-stimulating molecule produced by Efa was identified using liquid chromatography mass spectrometry (LC-MS). The proliferative effect of metabolites on CRC cells in vitro was assayed as well. The concentration of vascular endothelial growth factor A (VEGFA) and interleukin-8 (IL-8) was determined using enzyme-linked immunosorbent assay (ELISA). Tubular formation assay of human umbilical vein endothelial cells (HUVEC) and cell migration assay were applied to study angiogenesis. Additionally, western blot analysis was used to investigate key regulatory proteins involved in the angiogenesis pathway. Tumor growth was assessed using mouse models with two CRC cells and human colon cancer organoid.

**Results:**

Co-incubation with the conditioned medium of Efa increased the proliferation of cultured CRC cells. Biliverdin (BV) was determined as the key metabolite produced by Efa using LC-MS screening. BV promoted colony formation and cell proliferation and inhibited cell cycle arrest of cultured CRC cells. BV significantly increased the expression level of IL-8 and VEGFA by regulating the PI3K/AKT/mTOR signaling pathway, leading to the acceleration of angiogenesis in CRC. The up-regulation of proliferation and angiogenesis by BV were also confirmed in mice.

**Conclusion:**

In conclusion, BV, as the tumor-stimulating metabolite of Efa, generates proliferative and angiogenic effects on CRC, which is mainly mediated by the activation of PI3K/AKT/mTOR.

**Supplementary Information:**

The online version contains supplementary material available at 10.1186/s12967-023-03929-7.

## Background

Colorectal cancer (CRC) is the fourth leading cause of cancer death and the third most common cancer globally [1]. Despite certain improvements in screening and therapy [2, 3], the incidence and mortality of CRC remain high even in high-income countries. The 5 year survival rate of patients with advanced CRC is still less than 10% [4]. Therefore, novel therapeutic targets and molecular pathogenesis of CRC are highly warranted.

The correlation between microbiome and CRC occurrence and development is increasingly appreciated. Microbial dysbiosis plays an important role in CRC aetiology and modulates the pathological functions of CRC, such as cell proliferation, apoptosis and immune response [5, 6]. Current studies have shown that intestinal microbiota, such as *Clostridium*, *Bacteroides*, *Enterococcus*, and *Escherichia* genera [7], can facilitate colorectal carcinogenesis by directly interacting with host cancer cells, generating carcinogenic microbial metabolites, and secreting oncogenic virulence factors [8–10]. The gut microbiota can generate various metabolites and other small molecules, including secondary bile acids, short-chain fatty acids (SCFAs), indole and some amino acid metabolites. Therefore, the gut microbial metabolome is associated with the occurrence and development of CRC.

*Enterococcus faecalis* (Efa) is a gram-positive, facultative anaerobic symbiotic bacterium in the gastrointestinal tract and oral cavity. Current study has showed that the abundance of Efa increased in the stool and adjacent tissues of CRC patients [11]. In addition, Efa as a “driver bacteria” in the occurrence and development of CRC, can promote CRC by inducing inflammation and facilitate the accumulation of additional mutations and epigenetic changes [12–14]. However, the role of Efa in promoting the occurrence and development of CRC through its metabolites has not been clarified. Therefore, the functional role of Efa metabolites in driving intestinal tumorigenesis in vivo and in vitro was investigated in the present study.

## Materials and methods

### Bacterial strain and culture condition

Efa ATCC 29212 and *E. coli* ATCC 25922 were used in this study. The isolates were inoculated on Mueller-Hinton II agar (MHA) for 24 h at 37 ℃ and then suspended at the multiplicity of infection (MOI) 100:1 in RPMI 1640 medium (Gibco, Carlsbad, CA, USA). The isolates and cells were co-incubated and cultured continuously for 6 h at 37 ℃. Finally, the conditioned medium was obtained by centrifuging the bacteria culture medium at 6000 × g for 10 min and filtering with a 0.2 mm pore-size filter.

### Cell culture

Four commonly used CRC cell lines (HT29, HCT116, SW480, SW620) and one human normal colonic epithelial cell line NCM460 were obtained from ATCC. HT29 was highly differentiated, HCT116 and SW480 were CRC cell lines in situ, while SW620 was advanced lymph node metastatic CRC cell. HT29 was routinely cultured in McCOy’s 5A medium (Gibco, Carlsbad, CA, USA), HCT116 was cultured in Dulbecco’s Modified Eagle’s Medium (DMEM) (ibid.), while the other three cell lines were cultured in RPMI 1640 medium (ibid.), added with 10% (vol/vol) fetal bovine serum (FBS), and 1% penicillin/streptomycin in a humid atmosphere of 5% CO_2_.

The human umbilical vein endothelial cells (HUVECs) were bought from ATCC and cultured in DMEM, added with 1% penicillin/streptomycin and 10% FBS in a humid incubator containing 5% CO_2_ at 37 °C.

### Reagent

Biliverdin (BV) was purchased from J&K Scientific (B386400, Germany), which was dissolved in 0.1% DMSO (sigma-aldrich, USA) to 200 mM and then diluted to different concentrations with cell culture medium or phosphate-buffered saline (PBS) (Gibco, Carlsbad, CA, USA). To ensure appropriate control conditions, a reasonable quantity of DMSO was added to the medium in all the experiments using BV. All experiments were performed in a controlled way to avoid direct light exposure.

Inhibitors including PI3K inhibitor LY294002 (MedChemExpress, Monmouth Junction, NJ), and mTOR inhibitor Rapamycin (ibid.) were added to CRC cells at 10 μM and 100 μM for 48 h, respectively.

### Untargeted metabolomics by liquid chromatography mass spectrometry (LC–MS)

The untargeted metabolomics sequencing was conducted by Novogene Co., Ltd. (Beijing, China) to explore the changes in the relative abundance of metabolites of the conditioned medium after 6 h co-culture of NCM 460 with or without Efa (MOI 100:1). The sample (1 mL) was freeze-dried and resuspended by well vortex with prechilled 80% methanol, followed by incubation on ice for 5 min and centrifugation for 15 min at 4 °C and 15,000 ×g. The supernatant was diluted with LC-MS grade water to a final concentration of 53% methanol. Subsequently, the sample was transferred to a fresh Eppendorf tube, followed by centrifugation for 15 min at 4 °C and 15,000 ×g. Eventually, the supernatant was injected into the LC-MS/MS system (ThermoFisher, Germany). Then the metabolites were annotated using the KEGG database (https://www.genome.jp/kegg/pathway.html), HMDB database (https://hmdb.ca/metabolites) and LIPID Maps database (http://www.lipidmaps.org/).

Principal components analysis (PCA) was performed at the software metaX [15]. We applied univariate analysis (t-test) to calculate the statistical significance (P-value). The metabolites with variable important in projection (VIP) > 1 and P-value < 0.05 and fold change (FC) ≥ 2 or FC ≤ 0.5 were considered to be differential metabolites. For clustering heat maps, the data were normalized using z-scores of the intensity areas of differential metabolites and were ploted by Pheatmap package in R language. The functions of these metabolites and metabolic pathways were studied using the KEGG database. The metabolic pathways enrichment of differential metabolites was performed using the R package clusterProfiler (https://github.com/YuLab-SMU/clusterProfiler), when ratio were satisfied by x/n > y/N, metabolic pathway were considered as enrichment, when P-value of metabolic pathway < 0.05, metabolic pathway were considered as statistically significant enrichment.

### Cell growth assay

Cell viability was evaluated using the Cell Counting Kit-8 (MedChemExpress, Monmouth Junction, NJ). For the 96-well plate, 3000 cells were seeded in each well and directly treated with different concentrations of BV or bacterial conditioned medium. HCT116 and SW480 cells were treated with the bacterial conditioned medium for 4 consecutive days at the concentration of 20% (vol/vol). After each well was added with 10 μL CCK8 solution, the absorbance at 450 nm was determined per day at 37 °C after incubation for 2 h.

A colony formation assay was then performed. 500 cells were seeded in the 6-well plate, followed by changing the treatment medium every 2 days. After 10–14 days of culture, cells were stained with 0.1% crystal violet solution and fixed with 4% paraformaldehyde (Sigma, St. Louis, MO). Over 50 cells were counted in the colony. All experiments were conducted in triplicate three times.

### Cell cycle analysis

The treated cells were fixed with 70% ethanol for cell cycle analysis. After centrifugation (5 min, 1000 ×g) and resuspension, the cells were kept out of light and incubated with propidium iodide (PI) (Beyotime, Shanghai, China) for 30 min at room temperature. 10,000 cells were counted using CytoFlex LX (Beckman Coulter, USA), and the cell cycle profile was studied using FlowJo V10 software.

### Enzyme-linked immunosorbent assay (ELISA)

The protein levels of IL-8 and VEGFA in the treated cell culture supernatant, plasma and intracellular protein of nude mice were measured with sandwich ELISA kits (Proteintech Group, Wuhan, China, #KE00006 for IL-8; RayBiotech, Inc. Georgia, GA, USA, #ELH-VEGF-1 for VEGFA) according to the manufacturer’s instructions.

### Real-time quantitative PCR

Total RNA was extracted from cells by commercial kits (ES-RN001, Yishan Biotech, Shanghai, China). RNA concentration was measured with NanoDrop One Spectrophotometer (Thermo Scientific, USA). Complementary DNA (cDNA) was synthesized by reverse transcription of 1 μg total RNA from each sample using cDNA Synthesis kits (RR047A, Takara Bio Inc., Japan). Subsequently, the resultant cDNA was applied in real-time PCR on StepOnePlus^™^ real-time PCR instrument (Applied Biosystems, Foster, CA, USA) to evaluate the gene expression of IL-8 and VEGFA. The reactions were incubated in a 96-well plate at 95 °C for 1 min, followed by 40 cycles of 95 °C for 5 s and 60 °C for 30 s. GAPDH was used as an internal control. The following are the primer sequences: IL-8 forward, 5′-ACATACTCCAAACCTTTCCACC-3′ and reverse, 5′-AAAACTTCTCCACAACCCTCTG-3′; GAPDH forward, 5′-CTGGGCTACACTGAGCACC-3′ and reverse, 5′-AAGTGGTCGTTGAGGGCAATG-3′; VEGFA forward, 5′-ATGAACTTTCTGCTGTCTTGG-3′ and reverse 5′-TCACCGCCTCGGCTTGTCACA-3. All experiments were conducted independently three times. The gene expression of IL-8 and VEGFA was normalized to GAPDH and calculated based on the 2^-△△CT^ method.

### Western blot analysis

Total protein was extracted using RIPA lysis buffer containing phosphatase and protease inhibitors. Proteins were quantified using bicinchoninic acid (BCA) assay, and then separated with 10% SDS-PAGE gel and transferred to the PVDF membrane (Beyotime Institute of Biotechnology, Shanghai, China). The membrane was blocked with 5% BSA for 1 h and incubated overnight using rabbit antibodies to mTOR (1:1000, 2972S, Cell Signaling Technology), P-PI3K (1:1000, AF3242, Affinity Biosciences), P-mTOR (1:1000, 5536 T, Cell signaling Technology), PCNA (1:2000, 10205–2-AP, Proteintech Group), mouse antibodies to Actin (1:5000, 66009–1-Ig, Proteintech Group), PI3K (1:5000, 60225–1-Ig, Proteintech Group), AKT (1:2000, 60203–2-Ig, Proteintech Group), P-AKT (1:2000, 66444–1-Ig, Proteintech Group). Subsequently, HRP-conjugated secondary antibodies were treated, and the signal was measured using the enhanced chemiluminescence detection system (SH-523, Hangzhou, China). The intensity was analyzed by Image J software.

### Conditioned medium (CM)

CRC cells (1 × 10^6^) were cultured overnight in the 6-well plate, followed by changing the medium to fresh medium with or without BV and inhibitors in each well. The conditioned medium was gathered after 48 h, which was applied for tube formation assay of HUVEC, migration, and ELISA.

### Cell migration assay

HUVEC migration was evaluated using Cell Migration Assay Kit (BD Biosciences, NJ, USA). In the 24-well plate, the 8 μm pore upper chamber was added with 250 μl serum-free medium containing 2.5 × 10^4^ HUVEC, and the lower chamber was added with 750 μl conditioned supernatant. Cells were subsequently incubated at 37 ℃ for 24 h within the system. The migrated cells were stained with 0.1% crystal violet and fixed with 4% paraformaldehyde (Sigma, St. Louis, MO) after non-invading cells were removed with cotton swabs. Eventually, the bias was minimized using a microscope with 100X magnification to count the stained cells in at least three fields selected randomly.

### Endothelial tube formation assay

HUVEC (2 × 10^4^) was plated in the 96-well plate containing 50 μl Matrigel (BD Biosciences, Bedford, MA) and cultured at 37 °C for 4 h in the conditioned medium of 5% CO_2_. A microscope was used to photograph tubules and Image-Pro Plus software was used for assessment.

### CRC organoid model

The human tumor microenvironment was simulated by the culture of CRC organoids [16]. The appropriate concentrations of BV were added directly to the medium. Change the treatment medium every 3 days. A microscope was used to observe the growth of CRC organoids per day.

### In vivo* xenograft model*

Male nude mice (4–5 weeks, BALB/C) were obtained from Hangzhou Ziyuan Laboratory Animal Technology Company. All animal experiments received the approval of the Institutional Animal Care and Use Committee at the First Affiliated Hospital of Zhejiang University. 5 × 10^6^ HCT116 and SW480 were subcutaneously injected into the right side of mice. After 7 days of tumor injection, mice in the treatment group received 100 μl 50 μM BV once a day via peritumoral injection, while the control group received an equal amount of PBS with DMSO. Tumor volume was determined every 2 days and tumor weight was measured at the end, to detect the effect of BV on tumor growth.

### Immunohistochemistry (IHC)

All xenograft tumor specimens were fixed in 4% paraformaldehyde at 4 °C for 48 h and serially sectioned into 5 μm-thick sections. Then paraffin-embedded tissue chips were dried at 90 °C for 4 h, dewaxed in xylene, and then rehydrated in a series of graded ethanol solutions. Sodium citrate buffer (10 mM, pH 6.0) was used for antigen retrieval. 3% hydrogen peroxide solution was used to block endogenous peroxidase activity for the tissue sections which followed by rinsing with PBS for 15 min, and blocking with 3% BSA solution for 30 min. The sections were then incubated with the mouse anti-CD31 antibody (GB113151, Servicebio, 1:300) at 4 °C overnight, followed by incubation with HRP-labeled rabbit secondary antibody (GB23303, Servicebio, 1:200) at 37 °C for 50 min. Diaminobenzene was used as the chromogen, and hematoxylin was used as the nuclear counterstain. Sections were then dehydrated, cleared, and mounted. The grade of microvessel growth in tumor was assessed by the relative percentage of CD31-positive areas that was expressed as the microvessel density (MVD) in each high-power field.

### Immunofluorescence staining

The slices were fixed with 10% formalin and embedded in paraffin for immunofluorescence staining. Tissue slices from tumor areas were then deparaffinized and subjected to antigen retrieval using citrate buffer three times for 10 min each in a microwave oven, followed by sequential incubation in 30% hydrogen peroxide in methanol and blocking with 0.5% BSA for 30 min. Tissue slices were then incubated with the following primary antibodies: anti-CD31 (1:300, GB113151, Servicebio) and anti-ki67 (1:300, GB121141, Servicebio). The secondary antibodies used were Alexa Fluor CY3-conjugated anti-mouse (1:300, GB21301, Servicebio) and Alexa Fluor 488-conjugated anti-rabbit (1:400, GB25303, Servicebio). A microscope was used for imaging.

### Statistical analysis

Data were represented by mean ± SD. The difference between the two groups was compared using the Mann-Whitney U test or Student’s t test. The difference between multiple groups was compared by one-way variance analysis, and two-way variance analysis was used to determine the difference in cell viability. Results of statistical significance were indicated as follows: *P < 0.05; **P < 0.01; ***P < 0.001; ****P < 0.0001. All tests were conducted through Graph-Pad Prism 7.0 software (GraphPad Software, San Diego, CA).

## Results

### Efa conditioned medium promotes the viability of colon cancer cells

**Fig. 1:**
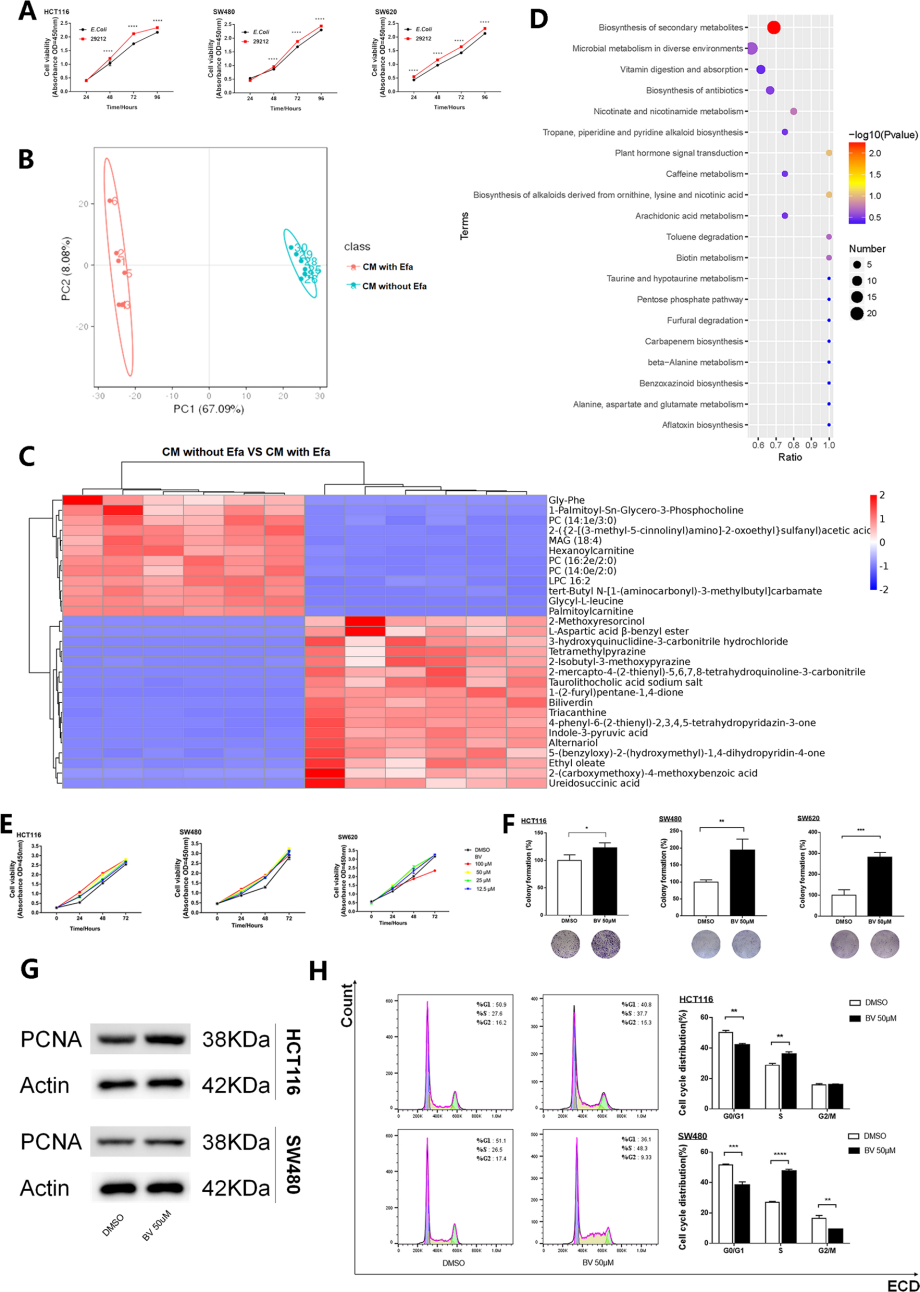
The conditioned medium and the metabolite of Efa promote the viability of CRC cells in vitro. **A** CCK-8 assay in CRC cells treated with the conditioned medium of EFa or E. coli ATCC 25922. **B**–**D** Metabolomics analysis of the conditioned medium (CM) with or without Efa. B: Principal Component Analysis (PCA) score plot of metabolomics. The orange dots represent the conditioned medium (CM) with Efa, and the blue dots represent the CM without Efa. C: Heatmap of differentially metabolites. The p-values were represented by a color scale from blue (relatively lower expression) to red (relatively higher expression). Each column represented individual sample, and each row represented a single metabolite. D: KEGG analysis for the various differentially expressed signal pathway. The dots represented various pathways. Pathways impact was represented by the area of each dots. The p-value was represented by a color scale from blue (relatively lower significance) to orange (relatively higher significance). **E**, **F** Proliferation ability of BV was determined by CCK8 and colony formation assay in CRC cells. **G** Proliferating cell nuclear antigen (PCNA) levels in CRC cells treated with or without BV was detected by western blot. **H** Flow cytometry showing the percentages of BV treated cells and control cells at different cell cycle phase. The histogram on the left is the control group, and the right is the BV treatment group. *, P < 0.05; **, P < 0.01; ***, P < 0.001; and ****, P < 0.0001

Colon cancer cells HCT116, SW480, and SW620 were co-cultured in the conditioned medium of Efa for 4 consecutive days. *E. coli* ATCC 25922 was used as bacterial control. The CCK8 assay indicated that compared to *E. coli*, the conditioned medium from Efa could accelerate the proliferation of colon cancer cells (Fig. 1A), indicating that Efa promotes colorectal carcinogenesis by bacteria-secreted molecules.

### Metabolite profiling of Efa

Changes in the relative abundance of metabolites were explored in the conditioned medium with or without Efa. Notably, principal component analysis (PCA) demonstrated excellent isolation between the two groups (Fig. 1B). Heatmap analysis showed that the expression level of BV and the biosynthesis of secondary metabolites were remarkably up-regulated in the conditioned medium of Efa compared to the normal control (Fig. 1C–D). The separate metabolomic analysis of Efa also found that BV was still a higher differential metabolite in Efa between different MOI (Additional file 4). And with the increase of Efa, more BV was metabolized (Additional file 5: Table S1).

### BV promotes the growth of CRC cells

As shown in Fig. 1E, CRC cells were cultured with BV at different concentrations (0, 12.5, 25, 50, 100 μM) at a certain time gradient (0, 24, 48, 72 h). The CCK-8 assay indicated that for both HCT116 and SW480, all concentrations of BV promoted cell proliferation. For SW620, except 100 μM, all the other concentrations of BV had a significant effect to elevate the viability of cells. BV at a concentration of 50 μM showed a stimulative effect on all CRC cell lines. The action time for 48 h and the working concentration of 50 μM were chosen for subsequent experiments. The colony formation assay and the boosted cell nuclear antigen protein levels further confirmed this growth-promoting effect (Fig. 1F–G). Moreover, treatment with BV increased the proportion of cells in the synthesis (S) and decreased G0/G1-phase cells in HCT116 and SW480 (Fig. 1H).

### BV promotes the progression of CRC cells by stimulating angiogenesis

**Fig. 2:**
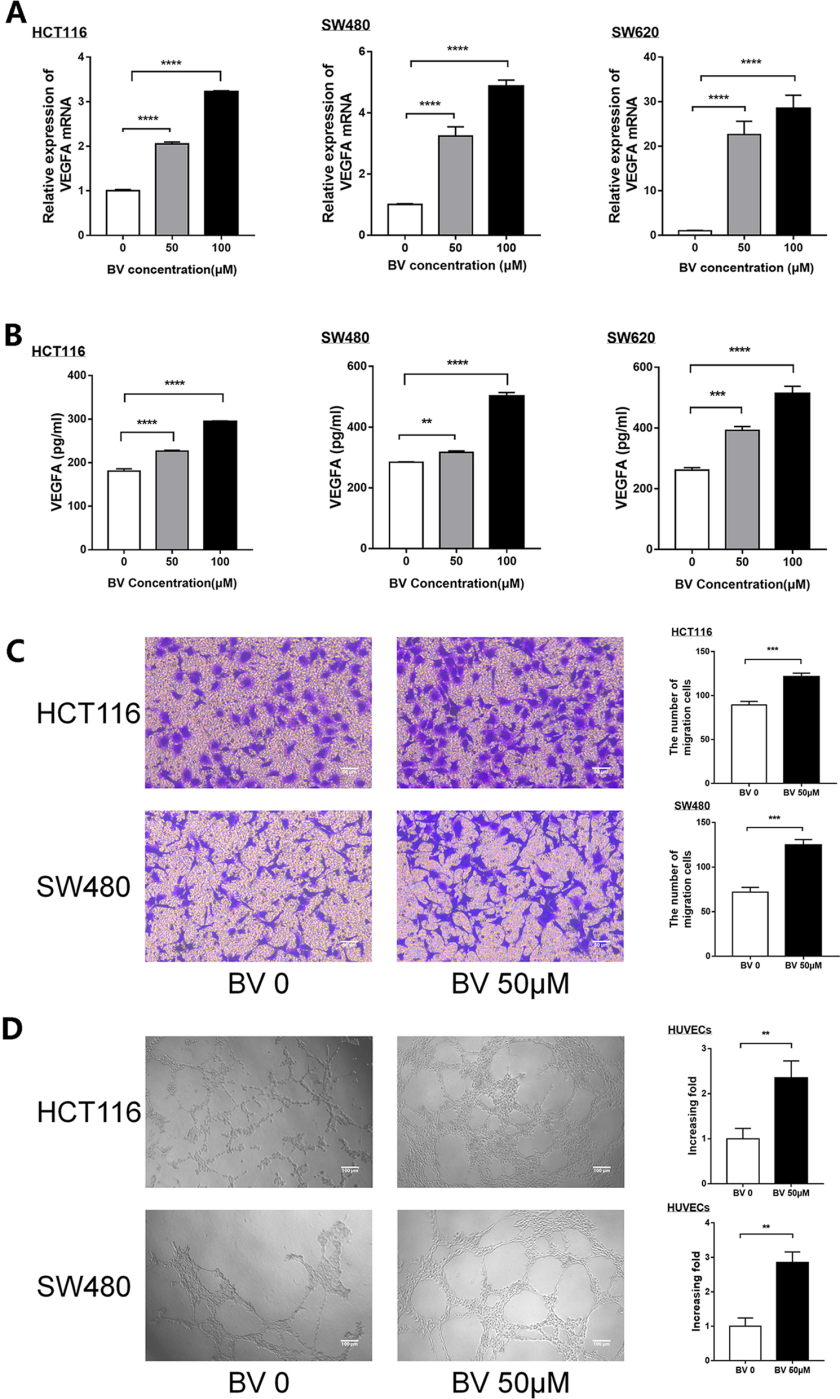
BV stimulates CRC angiogenesis by secreting VEGFA. **A**, **B** The expression of VEGFA was detected by RT-qPCR and ELISA in HCT116, SW480 and SW620 cells cultured with BV. **C** Cell migration in HUVECs were examined by transwell assays after HUVECs were plated and cocultured with the CM from HCT116 and SW480 treated with BV. One representative image from three reproducible experiments is shown. Scale bar, 50 μm. Migrated HUVEC numbers are shown in the bar graph. **D** Tubule formation of HUVECs was shown in representative images after co-incubating with the CM from BV treated HCT116 and SW480. Scale bar, 100 μm. The increasing folds of tube formation is shown in the bar graph. **P < 0.01, ***P < 0.001, ****, P < 0.0001

The mRNA expression of VEGFA was significantly up-regulated in a concentration-dependent manner in the three CRC cells cultured with BV (Fig. 2A). The ELISA assay showed that BV increased the protein concentration of VEGFA compared to the normal control (Fig. 2B). Interestingly, RT-qPCR and ELISA assay revealed that BV increased the expression of IL-8 in HCT116 and HT-29, but not in SW480 and SW620 (Additional file 1).

The migration of endothelial cells plays an important role in angiogenesis. Therefore, the effect of BV on the migration of HUVECs was assessed using the transwell cell assay, and the potential role of BV in angiogenesis was further elucidated by the tubular formation of HUVECs. CM of BV-treated CRC cells promoted the migration (Fig. 2C) and tube formation (Fig. 2D) of HUVECs. These findings demonstrated the important role of BV on the angiogenesis of HUVECs in CRC.

### BV promotes VEGFA expression and angiogenesis dependent on the PI3K/AKT/mTOR signaling pathway in CRC

**Fig. 3:**
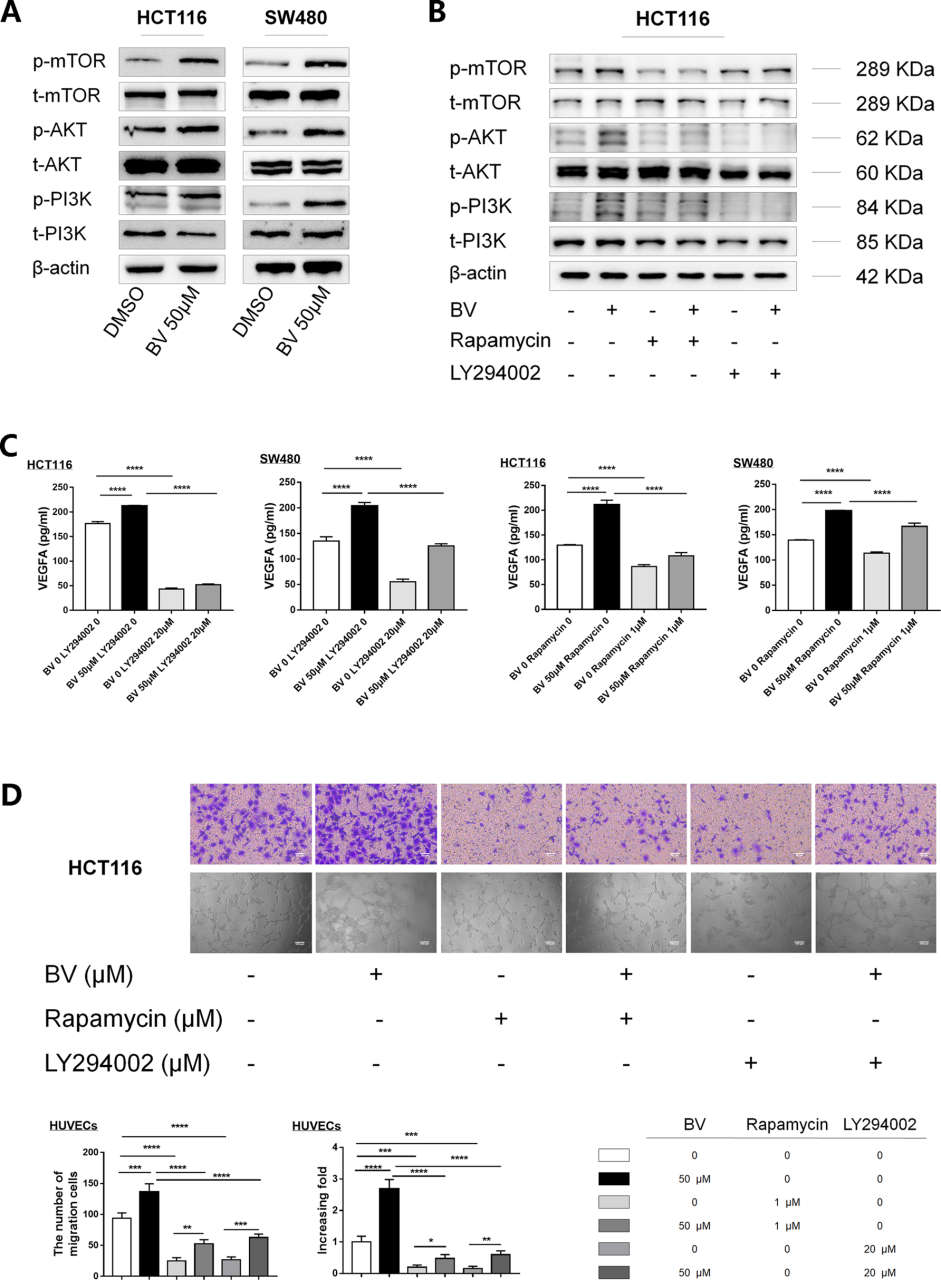
BV enhances VEGFA secretion and angiogenesis via activating PI3K/AKT/mTOR pathway. **A** The expression of PI3K/AKT/mTOR pathway members were detected by western blot in HCT116 and SW480 treated with or without BV. **B** Western blot analysis of PI3K/AKT/mTOR pathway members with or without the treatment of BV, LY294002 and Rapamycin in HCT116. **C** The concentration of VEGFA in the culture medium of control and BV treated HCT116 and SW480 cells with or without the absence of LY294002 and Rapamycin. **D** HUVEC migration and tube formation in representative images in the CM of HCT116 cocultured with BV with or without the absence of LY294002 and Rapamycin. Scale bar, 50 μm and 100 μm respectively. The migrated HUVEC numbers and increasing folds of tube formation are shown in the bar graph. *, P < 0.05; **, P < 0.01; ***, P < 0.001; and ****, P < 0.0001

The changing trend of signal molecules including t-mTOR, p-mTOR, t-AKT, p-AKT, t-PI3K, p-PI3K indicated that the PI3K/AKT/mTOR signal pathway was activated in HCT116 and SW480 cells treated with BV (Fig. 3A). At the same time, the downstream signaling pathway activated by BV was prevented by treatment with mTOR inhibitor Rapamycin and PI3K inhibitor LY294002 in the two cell lines respectively (Fig. 3B and Additional file 2A). After treatment with Rapamycin and LY294002 by ELISA, the concentrations of VEGFA and IL-8 were examined in the culture medium. The secretion of VEGFA (Fig. 3C) and IL-8 (Additional file 2B) was reduced by both Rapamycin and LY294002. Thus, BV could positively promote VEGFA expression by the PI3K/AKT/mTOR pathway.

The migration and tube formation of HUVECs treated with CM of BV-treated CRC cells (HCT116 and SW480) with or without inhibitors were examined to further investigate the role of the PI3K/AKT/mTOR signaling pathway in the tumor angiogenesis mediated by BV. As expected, the addition of LY294002 and Rapamycin significantly reversed the increased proangiogenic capacity of HUVECs treated with CM of BV-treated HCT116 (Fig. 3D) and SW480 (Additional file 2C).

### BV promotes proliferation and angiogenesis in vivo and human colon cancer organoid

**Fig. 4:**
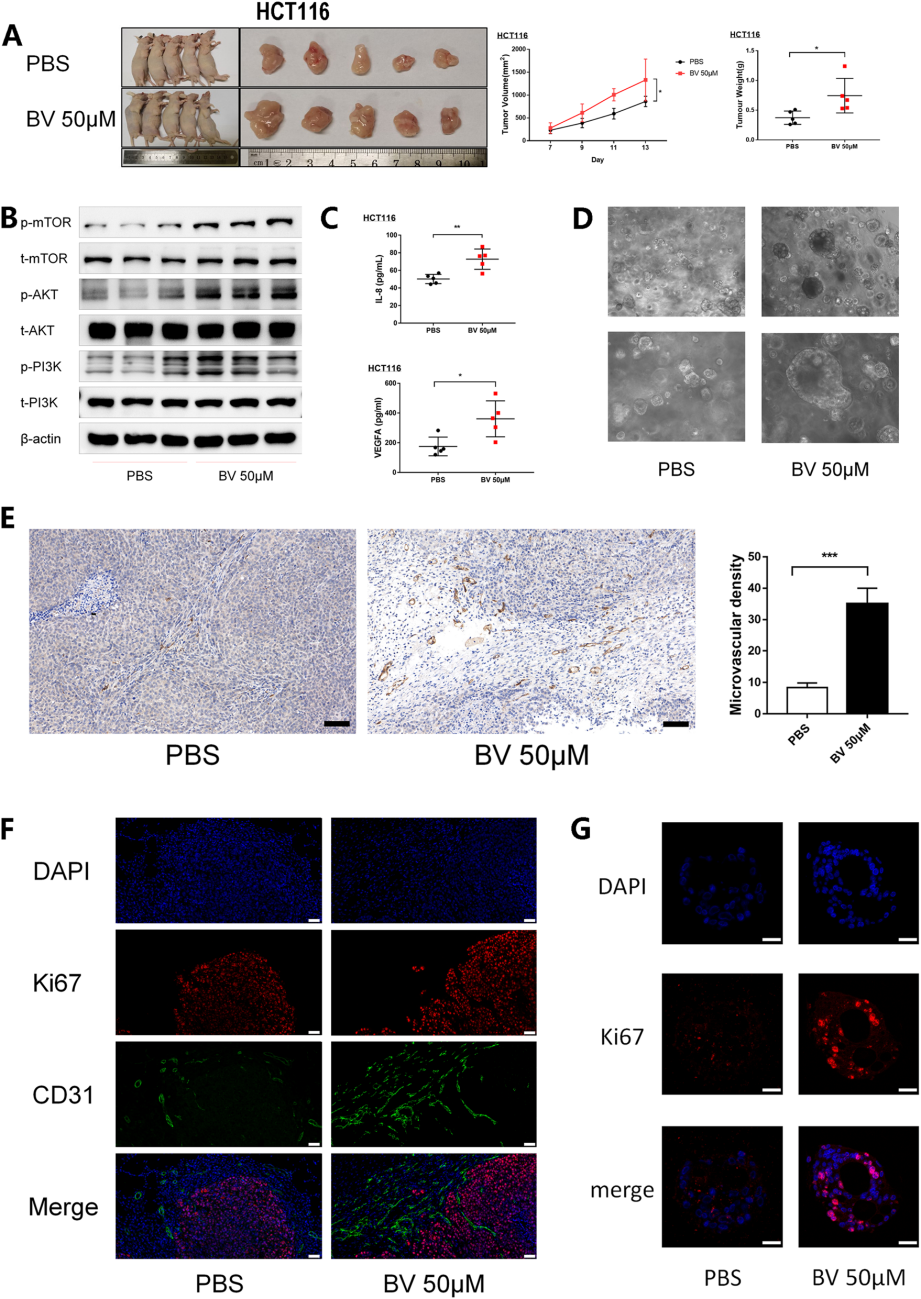
BV promotes proliferation and angiogenesis in vivo. **A** Representative images of subcutaneous tumors in nude mice injected HCT116 cells treated with or without BV. Both the volume and weight of subcutaneous tumor were shown in the right panel. **B** PI3K/AKT/mTOR pathway was activated in mice tumor by western blot analysis. **C** The concentration of IL-8 and VEGFA in mice tumors were detected by ELISA. **D** The growth of human colon cancer organoids was assessed after 10 days of BV treatment or not. **E** IHC analysis demonstrated the expression of CD31 in subcutaneous tumors of nude mice. Bars of the right panel represent the microvascular density. Scale bar represents 100 μm. **F**, **G** The expression of Ki67 and CD31 in subcutaneous tumors of nude mice and organoid models were assessed by immunofluorescence staining. Representative images were shown. Scale bar represents 50 μm and 20 μm, respectively. *, P < 0.05; **, P < 0.01; ***, P < 0.001

**Fig. 5:**
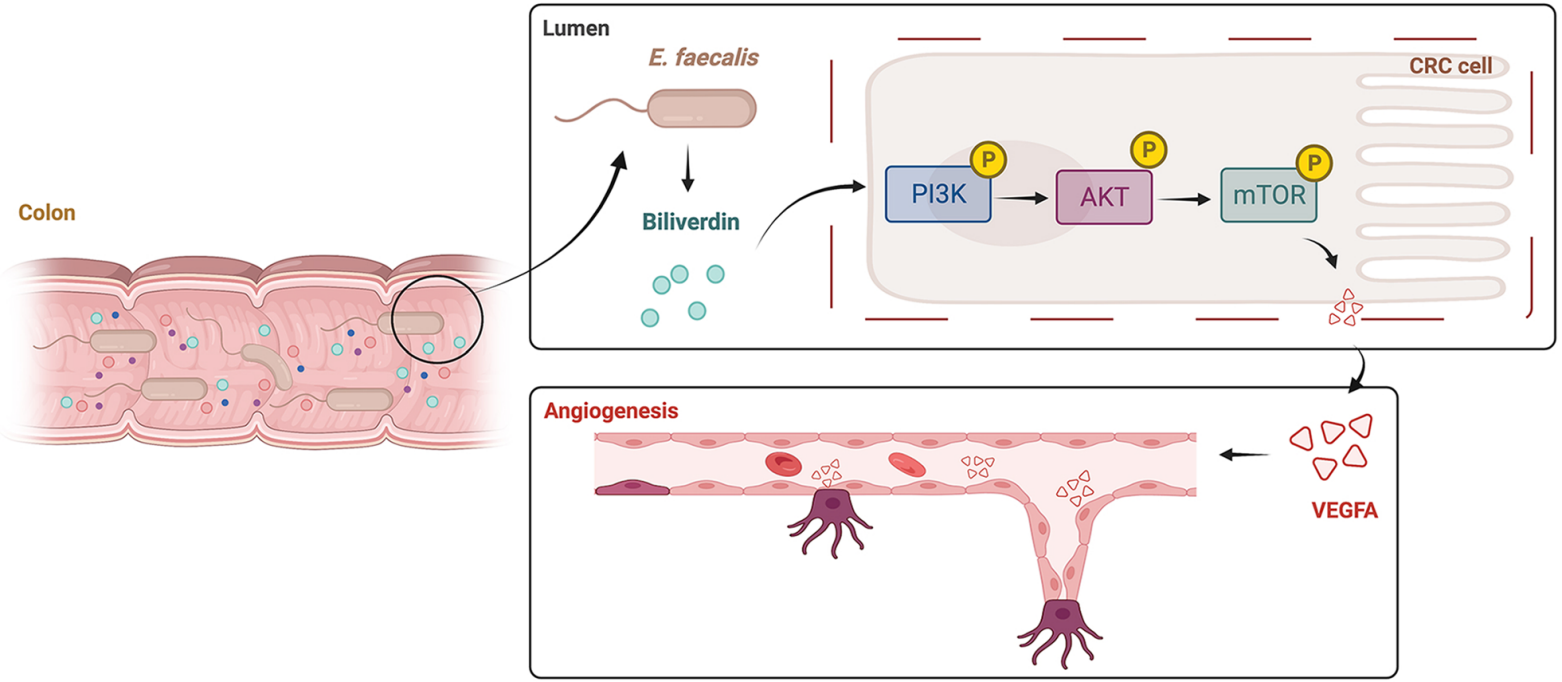
Schematic model of Efa/BV/PI3K/AKT/mTOR/VEGFA axis in CRC. Efa promotes colon tumorigenesis by the secretion of BV. BV promotes growth and angiogenesis in CRC by regulating PI3K/AKT/mTOR/VEGFA pathways

Xenograft models of HCT116 and SW480 were developed in nude mice to verify the mechanism in vivo. After peritumoral administration of BV, the growth rate of xenograft was higher than the control (Fig. 4A and Additional file 3A), which was also validated in the organoid model (Fig. 4D). Western blot analysis showed that the PI3K/AKT/mTOR signaling pathway was activated in subcutaneous tumors of nude mice treated with BV (Fig. 4B and Additional file 3B). More importantly, BV treatment elevated the expression of VEGFA (Fig. 4C) and IL-8 (Additional file 3C) in tumors (Fig. 5).

The expression of Ki67 and CD31 proteins was detected using immunofluorescence and immunohistochemical staining in subcutaneous tumors and organoid models. Results indicated that microvascular density shown by CD31 expression and Ki67 protein were higher in BV-treated models (Fig. 4E–G and Additional file 3D–E). Taken together, BV could promote the progression of CRC by by promoting proliferation and stimulating angiogenesis in vivo.

## Discussion

Colonic bacteria release specific metabolites into the colonic lumen through multiple metabolic pathways [10]. Metabolites may be a more robust clinical endpoint than microbial taxonomy, as they are the output of all combined microbial functions. Given that Efa up-regulates the expression of IL-8 and VEGFA in CRC cells, and that the conditioned medium of Efa promotes the viability of CRC cells, it can be hypothesized that Efa may perform this function through its metabolites. In this study, an untargeted metabolomic analysis was used to investigate the specific metabolites of Efa. Then, BV was screened according to the enriched metabolic pathways and differential metabolites with a statistical difference. In eukaryotes, BV is a product of the heme catabolic pathway, eukaryotic heme oxygenases (HOs) transfer heme to BV, iron and CO via three continuous oxygenation steps [17]. Similarly, heme oxygenase are also widespread found in prokaryotes and has a similar effect. The major function of bacterial heme oxygenase is to obtain iron in prokaryotic pathogens [18]. Iron is essential for bacterial growth and successful colonization of the host [19, 20]. Due to the absence of genes encoding homologues of mammalian biliverdin reductase (BVR) in bacteria, BV seems unlikely to be further metabolized to bilirubin (BR). Therefore, the production of BV in pathogens may be just a waste and then transported out of the cell [17]. But in human, BV have previously been considered to act as a natural antioxidant [21], protecting lipids from reactive oxygen species (ROS) as part of the biliverdin-bilirubin cycle [22]. At present, some studies have reported the beneficial effects of BV in biomedicine, such as reducing ischemia reperfusion injury of multiple organs [23, 24], inhibiting endotoxin-induced inflammatory response [25], and suppressing Porcine reproductive and respiratory syndrome (PRRSV) infection [26]. However, BV has also been investigated as a specific plasma metabolite biomarker in male patients with major depressive disorder [27]. Thus, the potential mechanism for the impact of BV on CRC cells was identified in this study, focusing on the impact of BV on angiogenesis, cell cycle, and proliferation.

In this study, BV, as the metabolite of Efa, promoted the progression of CRC. The viability of CRC cells was up-regulated by the conditioned medium from Efa and BV. Moreover, BV significantly increased tumor weight and tumor volume. This is the first study to characterize BV, the metabolite of Efa, as a tumor-promoting molecule. Further mechanism research indicated the critical participation of its angiogenesis, which was regulated by the PI3K/AKT/mTOR/VEGFA signaling pathways in CRC cells.

IL-8 is a pro-inflammatory chemokine that binds to two G-protein coupled receptors, CXCR1 and CXCR2, which is responsible for attracting neutrophils to sites of injury and inflammation [28]. IL-8 has multiple pro-tumorigenic functions in the context of tumors, such as modifying the composition of the tumor microenvironment (TME), affecting tumor cells themselves, exciting the transformation or proliferation of tumor cells into a mesenchymal or migratory phenotype, recruiting more immunosuppressive cells to tumors, and increasing tumor angiogenesis [29]. In addition, it has been reported that an increased level of IL-8 is related to the development of resistance to treatment and poor prognosis in many cancers [30, 31]. Interestingly, this study found that although the expression of IL-8 was elevated in HCT116 and HT29, but not in SW480 and SW620. Considering that HT29 and HCT116 are early CRC cells, it is assumed that BV may simultaneously play an important pro-inflammatory role in early CRC.

Angiogenesis activation is a fundamental mark of cancer, which is necessary for the growth and metastasis of invasive tumors [32]. Early in the development of invasive cancer, the switch of angiogenesis turns on through the domination of proangiogenic factors [33]. Tumor angiogenesis is mainly regulated by growth factors and cytokines generated from multiple cell types [34]. Previous studies have shown that VEGFA expression is related to poor clinical prognosis and is increased in CRC clinical tissues [35]. VEGFA, as an endothelial cell-specific mitogen, induces pathological and physiological angiogenesis through the activation of various signaling pathways that enhance vascular permeability and improve the growth, migration, and differentiation of endothelial cells [36, 37]. Thus, VEGFA has become a major target for anti-angiogenic drugs, which play a key role in CRC angiogenesis [38]. Consistent with the fact that VEGF production through dependent and independent pathways of hypoxia-inducible factor 1 (HIF-1) is promoted by stimulating the PI3K/AKT pathway in tumor cells [39], the PI3K/AKT pathway also contributes to the production of other angiogenic factors, including angiopoietin and nitric oxide. The cell proliferation and angiogenesis mediated by VEGF can be prevented through inhibition of the mTOR pathway by reducing the production and secretion of VEGF [40, 41]. Results of this study found that the expression of VEGFA was up-regulated through BV treatment on CRC cells, with a positive influence of the conditioned medium from BV-treated CRC cells on the angiogenesis of HUVECs, suggesting that BV facilitates the formation of nascent blood vessels by increasing the production and secretion of VEGFA. Furthermore, the results demonstrated that PI3K/AKT/mTOR could play a key role in inducing the angiogenesis of CRC. The administration of LY294002 and Rapamycin reversed the effect of the conditioned medium from BV-treated CRC cells on the angiogenesis of HUVECs. For an in vivo CRC tumor model, it was found that BV showed a significant increase in tumor growth as early as day 2 after treatment. Moreover, the expression of CD31, a specific and sensitive endothelial marker of microvessel density (MVD), was found to have a positive association with BV treatment. At the same time, this study also verified that BV facilitated the increase of IL8 and VEGF secretion by activating the PI3K/AKT/mTOR signaling pathway in tumor. Taken together, these results explicitly illustrate the important role of BV in the angiogenesis of CRC.

Although this study was performed with cell lines, CRC organoid, and mouse models of CRC, further additional interventional studies are needed in humans to validate these findings.

## Conclusion

In summary, Efa can promote colonic tumorigenesis by secretion of BV. BV is able to up-regulate IL-8 and VEGFA by the PI3K/AKT/mTOR signaling pathway, ultimately forming an angiogenic phenotype that stimulates the progression of CRC. Therefore, BV could be used as a novel biomarker for CRC tumorigenesis.

## Supplementary Information

**Additional file 1: **BV promotes IL-8 secretion in HCT116 and HT-29. The expression of IL-8 was detected by RT-qPCR and ELISA in HCT116, HT-29, SW480 and SW620 cells cultured with BV.

**Additional file 2: **BV enhances IL-8 secretion and angiogenesis via activating PI3K/AKT/mTOR pathway. (A) Western blot analysis of PI3K/AKT/mTOR pathway with or without the treatment of BV, LY294002 andRapamycin in SW480. (B) The concentration of IL-8 in the culture medium of control and BV treated HCT116, HT-29, SW480 and SW620 cells with or without the absence of LY294002 and Rapamycin. (C) HUVEC migration and tube formation in representative images in the CM of SW480 cocultured with BV with or without the absence of LY294002 and Rapamycin. Scale bar, 50μm and 100μm respectively. The migrated HUVEC numbers and increasing folds of tube formation are shown in the bar graph. *, P < 0.05; **, P < 0.01; ***, P < 0.001; and ****, P < 0.0001.

**Additional file 3: **BV promotes proliferation and angiogenesis in xenograft models of SW480. (A) Representative images of subcutaneous tumors in nude mice injected SW480 cells treated with or without BV. Both the volume and weight of subcutaneous tumor were shown in the right panel. (B) PI3K/AKT/mTOR pathway was detected in mice tumor by western blot analysis. (C) The concentration of IL-8 and VEGFA in mice tumors were detected by ELISA. (D) The expression of Ki67 and CD31 in subcutaneous tumors of nude mice were assessed by immunofluorescence staining. Representative images were shown. Scale bar represents 50μm. (E) IHC analysis demonstrated the expression of CD31 in subcutaneous tumors of nude mice. Bars of the right panel represent the microvascular density. Scale bar represents 100μm. **, P < 0.01.

**Additional file 4: **Metabolomics analysis of Efa with two different MOI (100:1 and 1:1). A: PCA score plot of metabolomics. The orange dots represent Efa in MOI 100:1, and the blue dots represent Efa in MOI 1:1. B: KEGG analysis for the various differentially expressed signal pathway. The dots represented various pathways. Pathways impact was represented by the area of each dots. The p-value was represented by a color scale from blue (relatively lower significance) to orange (relatively higher significance). C: Heatmap of differentially metabolites. The p-values were represented by a color scale from blue (relatively lower expression) to red (relatively higher expression). Each column represented individual sample, and each row represented a single metabolite. The left panel is Efa with MOI 1:1, and the right part is Efa with MOI 100:1.

**Additional file 5: Table S1.** The relative quantitative differences of BV in Efa with two different MOI (100:1 and 1:1).

## Data Availability

All data generated was showed in this manuscript. The datasets used and/or analysed during the current study are available from the corresponding author on reasonable request.

## Abbreviations

CRC: Colorectal cancer
Efa: *Enterococcus faecalis*
SCFAs: Short-chain fatty acids
MOI: Multiplicity of infection
HUVECs: Human umbilical vein endothelial cells
BV: Biliverdin
PBS: Phosphate-buffered saline
LC-MS: Liquid chromatography mass spectrometry
VEGFA: Vascular endothelial growth factor A
IL-8: Interleukin-8
ELISA: Enzyme-linked immunosorbent assay
PCA: Principal component analysis
CM: Conditioned medium
MVD: Microvessel density

## References

[1] Siegel RL, Miller KD, Jemal A (2019). Cancer statistics, 2019. CA Cancer J Clin.

[2] Ganesh K, Stadler ZK, Cercek A, Mendelsohn RB, Shia J, Segal NH (2019). Immunotherapy in colorectal cancer: rationale, challenges and potential. Nat Rev Gastroenterol Hepatol.

[3] Rotte A (2019). Combination of CTLA-4 and PD-1 blockers for treatment of cancer. J Exp Clin Cancer Res.

[4] Cidon EU (2010). The challenge of metastatic colorectal cancer. Clin Med Insights Oncol.

[5] Shen XJ, Rawls JF, Randall T, Burcal L, Mpande CN, Jenkins N (2010). Molecular characterization of mucosal adherent bacteria and associations with colorectal adenomas. Gut Microbes.

[6] Zackular JP, Baxter NT, Iverson KD, Sadler WD, Petrosino JF, Chen GY (2013). The gut microbiome modulates colon tumorigenesis. mBio.

[7] Wong SH, Yu J (2019). Gut microbiota in colorectal cancer: mechanisms of action and clinical applications. Nat Rev Gastroenterol Hepatol.

[8] Humphries JD, Byron A, Humphries MJ (2006). Integrin ligands at a glance. J Cell Sci.

[9] Rubinstein MR, Wang X, Liu W, Hao Y, Cai G, Han YW (2013). Fusobacterium nucleatum promotes colorectal carcinogenesis by modulating E-cadherin/beta-catenin signaling via its FadA adhesin. Cell Host Microbe.

[10] Louis P, Hold GL, Flint HJ (2014). The gut microbiota, bacterial metabolites and colorectal cancer. Nat Rev Microbiol.

[11] Balamurugan R, Rajendiran E, George S, Samuel GV, Ramakrishna BS (2008). Real-time polymerase chain reaction quantification of specific butyrate-producing bacteria, desulfovibrio and enterococcus faecalis in the feces of patients with colorectal cancer. J Gastroenterol Hepatol.

[12] Balish E, Warner T (2002). Enterococcus faecalis induces inflammatory bowel disease in interleukin-10 knockout mice. Am J Pathol.

[13] Wang X, Allen TD, Yang Y, Moore DR, Huycke MM (2013). Cyclooxygenase-2 generates the endogenous mutagen trans-4-hydroxy-2-nonenal in enterococcus faecalis-infected macrophages. Cancer Prev Res.

[14] Wang X, Huycke MM (2007). Extracellular superoxide production by enterococcus faecalis promotes chromosomal instability in mammalian cells. Gastroenterology.

[15] Wen B, Mei ZL, Zeng CW, Liu SQ (2017). MetaX: a flexible and comprehensive software for processing metabolomics data. Bmc Bioinform.

[16] Chen X, Huang Y, Liu J, Lin W, Chen C, Chen Y (2022). EXOSC5 promotes proliferation of gastric cancer through regulating AKT/STAT3 signaling pathways. J Cancer.

[17] Wilks A (2002). Heme oxygenase: evolution, structure, and mechanism. Antioxid Redox Sign.

[18] Frankenberg-Dinkel N (2004). Bacterial heme oxygenases. Antioxid Redox Signal.

[19] Sheldon JR, Laakso HA, Heinrichs DE (2016). Iron acquisition strategies of bacterial pathogens. Microbiol Spectr.

[20] Palmer LD, Skaar EP (2016). Transition metals and virulence in bacteria. Annu Rev Genet.

[21] Seok J, Ko YJ, Lee ME, Hyeon JE, Han SO (2019). Systems metabolic engineering of corynebacterium glutamicum for the bioproduction of biliverdin via protoporphyrin independent pathway. J Biol Eng.

[22] Sedlak TW, Saleh M, Higginson DS, Paul BD, Juluri KR, Snyder SH (2009). Bilirubin and glutathione have complementary antioxidant and cytoprotective roles. P Natl Acad Sci USA.

[23] Li JJ, Jiang HY, Peng PH, Zhang Q, Bai WY, Yang Y (2022). Biliverdin modulates the long non-coding RNA H19/microRNA-181b-5p/ endothelial cell specific molecule 1 axis to alleviate cerebral ischemia reperfusion injury. Biomed Pharmacother.

[24] Andria B, Bracco A, Attanasio C, Castaldo S, Cerrito MG, Cozzolino S (2013). Biliverdin protects against liver ischemia reperfusion injury in swine. PLoS ONE.

[25] Wegiel B, Gallo D, Csizmadia E, Roger T, Kaczmarek E, Harris C (2011). Biliverdin inhibits toll-like receptor-4 (TLR4) expression through nitric oxide-dependent nuclear translocation of biliverdin reductase. P Natl Acad Sci USA.

[26] Zhang AK, Duan H, Li N, Zhao LJ, Pu FX, Huang BC (2017). Heme oxygenase-1 metabolite biliverdin, not iron, inhibits porcine reproductive and respiratory syndrome virus replication. Free Radical Bio Med.

[27] Jiang YL, Qin MC, Teng T, Li XM, Yu Y, Wang J (2022). Identification of sex-specific plasma biomarkers using metabolomics for major depressive disorder in children and adolescents. Front Psychiatry.

[28] Waugh DJ, Wilson C (2008). The interleukin-8 pathway in cancer. Clin Cancer Res.

[29] Liu Q, Li A, Tian Y, Wu JD, Liu Y, Li T (2016). The CXCL8-CXCR1/2 pathways in cancer. Cytokine Growth Factor Rev.

[30] Cheng Y, Ma XL, Wei YQ, Wei XW (2019). Potential roles and targeted therapy of the CXCLs/CXCR2 axis in cancer and inflammatory diseases. Biochim Biophys Acta Rev Cancer.

[31] Fousek K, Horn LA, Palena C (2021). Interleukin-8: a chemokine at the intersection of cancer plasticity, angiogenesis, and immune suppression. Pharmacol Ther.

[32] Hanahan D, Weinberg RA (2011). Hallmarks of cancer: the next generation. Cell.

[33] Baeriswyl V, Christofori G (2009). The angiogenic switch in carcinogenesis. Semin Cancer Biol.

[34] de Palma M, Biziato D, Petrova TV (2017). Microenvironmental regulation of tumour angiogenesis. Nat Rev Cancer.

[35] Takahashi Y, Kitadai Y, Bucana CD, Cleary KR, Ellis LM (1995). Expression of vascular endothelial growth-factor and its receptor, Kdr, correlates with vascularity, metastasis, and proliferation of human colon-cancer. Cancer Res.

[36] Leung DW, Cachianes G, Kuang WJ, Goeddel DV, Ferrara N (1989). Vascular endothelial growth-factor is a secreted angiogenic mitogen. Science.

[37] Tischer E, Gospodarowicz D, Mitchell R, Silva M, Schilling J, Lau K (1989). Vascular endothelial growth factor: a new member of the platelet-derived growth factor gene family. Biochem Biophys Res Commun.

[38] Jayson GC, Kerbel R, Ellis LM, Harris AL (2016). Antiangiogenic therapy in oncology: current status and future directions. Lancet.

[39] Karar J, Maity A (2011). PI3K/AKT/mTOR pathway in angiogenesis. Front Mol Neurosci.

[40] Ji L, Wu M, Li Z (2018). Rutacecarpine inhibits angiogenesis by targeting the VEGFR2 and VEGFR2-mediated Akt/mTOR/p70s6k signaling pathway. Molecules.

[41] Wang M, Xu Y, Wen GZ, Wang Q, Yuan SM (2019). Rapamycin suppresses angiogenesis and lymphangiogenesis in melanoma by downregulating VEGF-A/VEGFR-2 and VEGF-C/VEGFR-3 expression. Onco Targets Ther.

