# Supplementary material for: Enterococcus faecalis promotes the progression of colorectal cancer via its metabolite: biliverdin
Source: J Transl Med. 2023 Feb 2;21:72. doi: 10.1186/s12967-023-03929-7 (PMC9896694; doi:10.1186/s12967-023-03929-7)
Supplement: Supplementary file 1 — Additional file 1: BV promotes IL-8 secretion in HCT116 and HT-29. The expression of IL-8 was detected by RT-qPCR and ELISA in HCT116, HT-29, SW480 and SW620 cells cultured with BV. [file 12967_2023_3929_MOESM1_ESM.pdf]

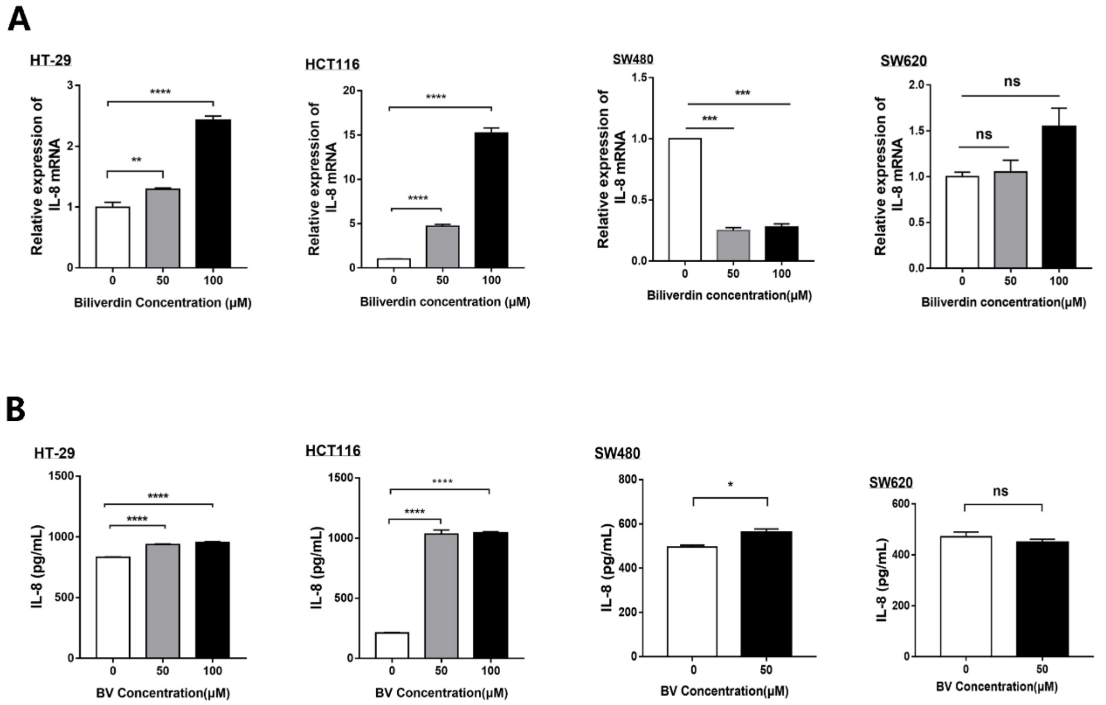

Additional file 1: BV promotes IL-8 secretion in HCT116 and HT-29

The expression of IL-8 was detected by RT-qPCR and ELISA in HCT116, HT-29, SW480 and SW620 cells cultured with BV.
