# Supplementary figures and images for: Enterococcus faecalis promotes the progression of colorectal cancer via its metabolite: biliverdin
Source: J Transl Med. 2023 Feb 2;21:72. doi: 10.1186/s12967-023-03929-7 (PMC9896694; doi:10.1186/s12967-023-03929-7)

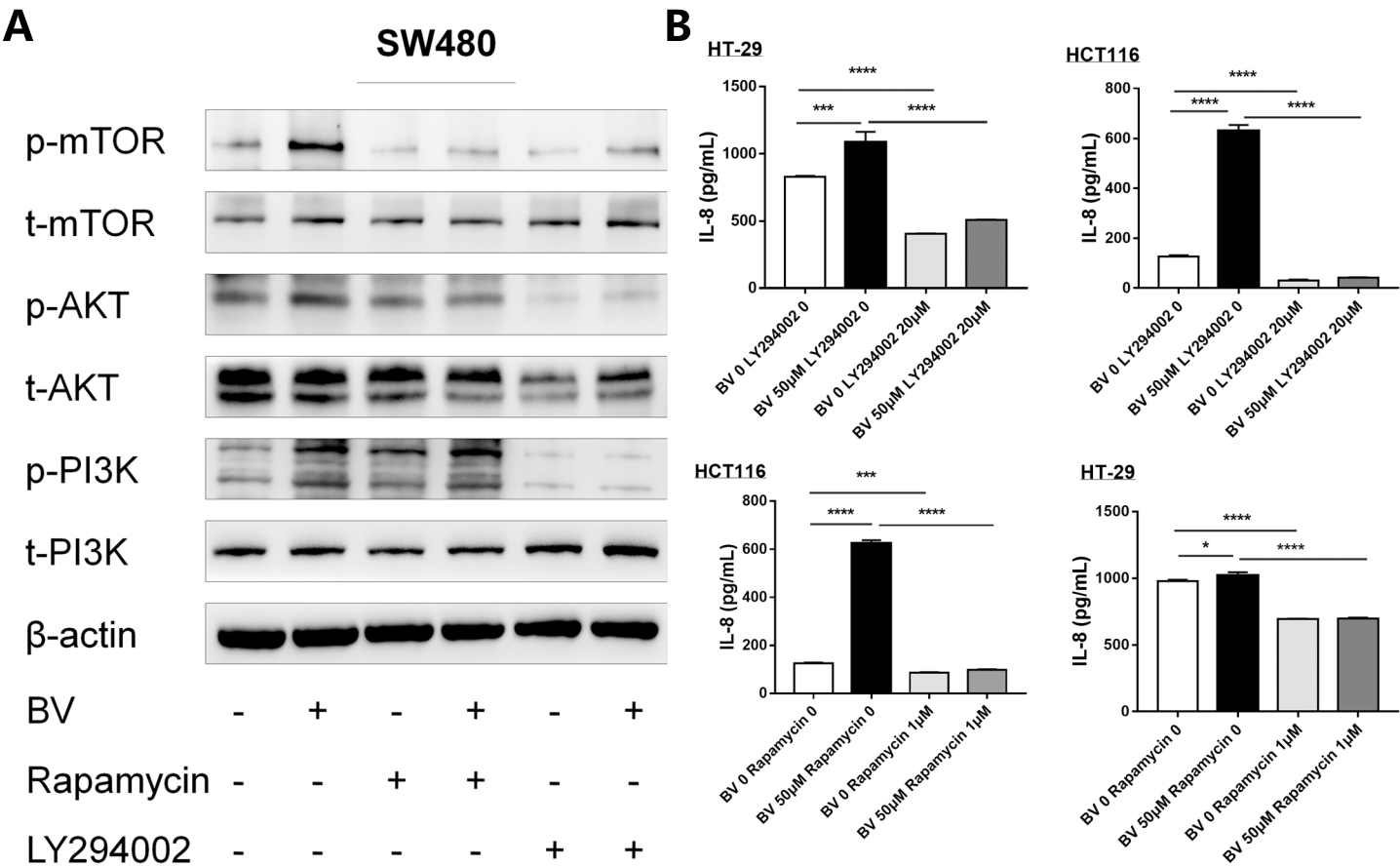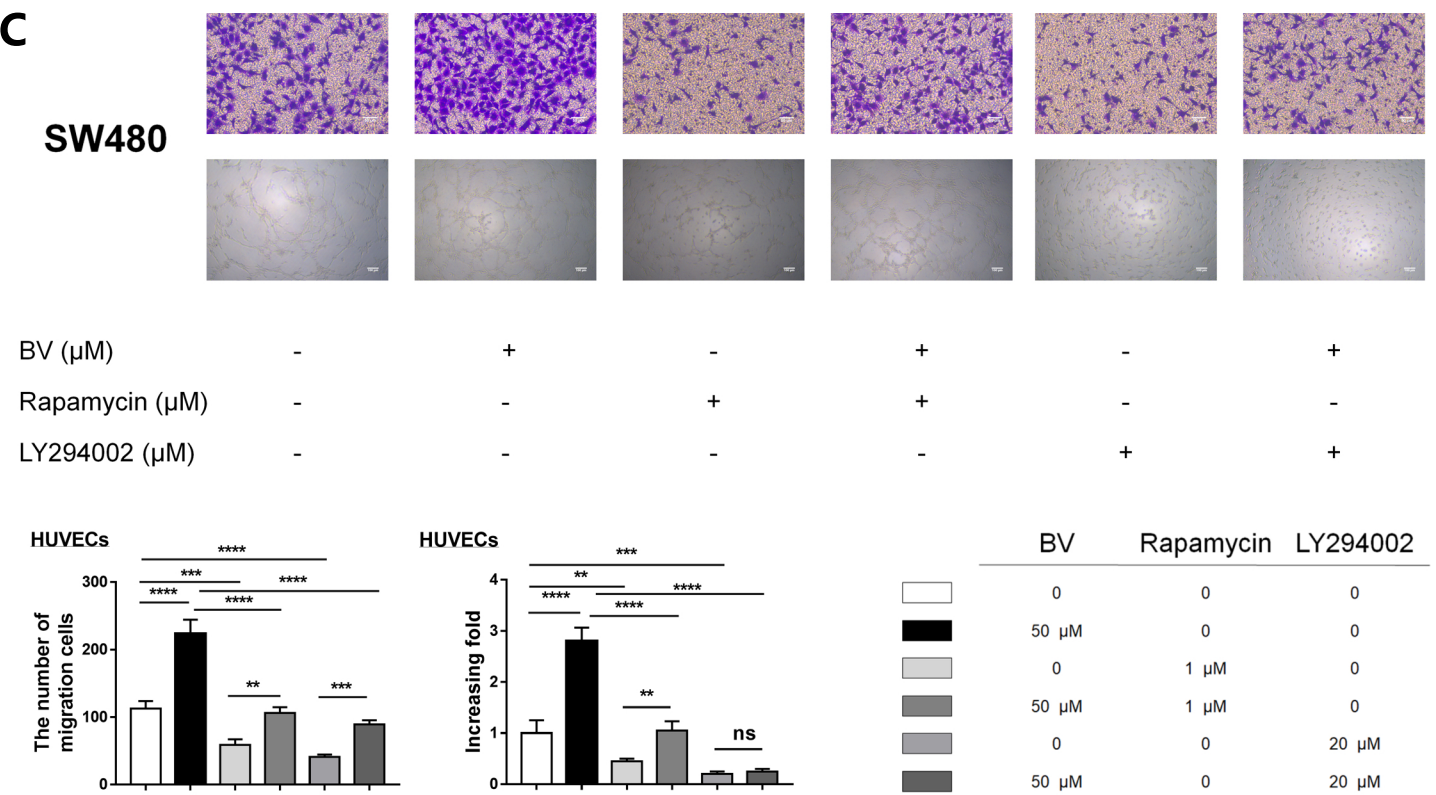

Supplement: Supplementary file 2 — Additional file 2: BV enhances IL-8 secretion and angiogenesis via activating PI3K/AKT/mTOR pathway. (A) Western blot analysis of PI3K/AKT/mTOR pathway with or without the treatment of BV, LY294002 andRapamycin in SW480. (B) The concentration of IL-8 in the culture medium of control and BV treated HCT116, HT-29, SW480 and SW620 cells with or without the absence of LY294002 and Rapamycin. (C) HUVEC migration and tube formation in representative images in the CM of SW480 cocultured with BV with or without the absence of LY294002 and Rapamycin. Scale bar, 50μm and 100μm respectively. The migrated HUVEC numbers and increasing folds of tube formation are shown in the bar graph. *, P < 0.05; **, P < 0.01; ***, P < 0.001; and ****, P < 0.0001. [file 12967_2023_3929_MOESM2_ESM.pdf]

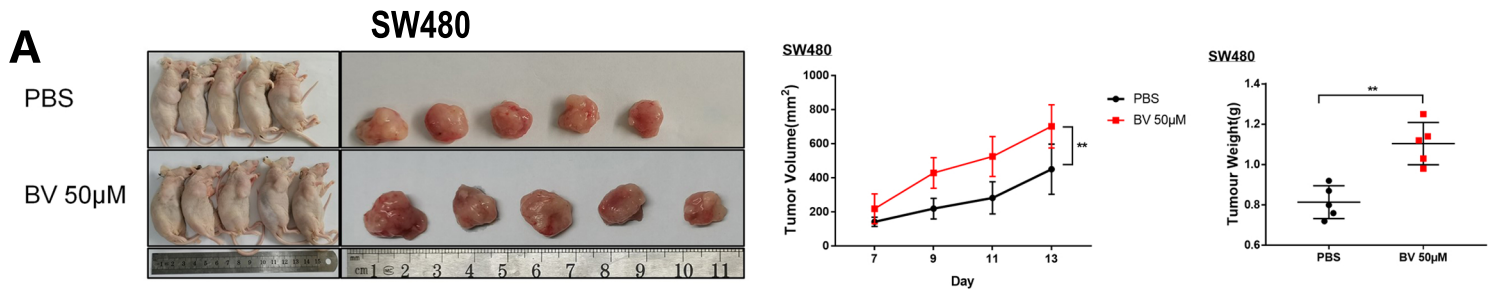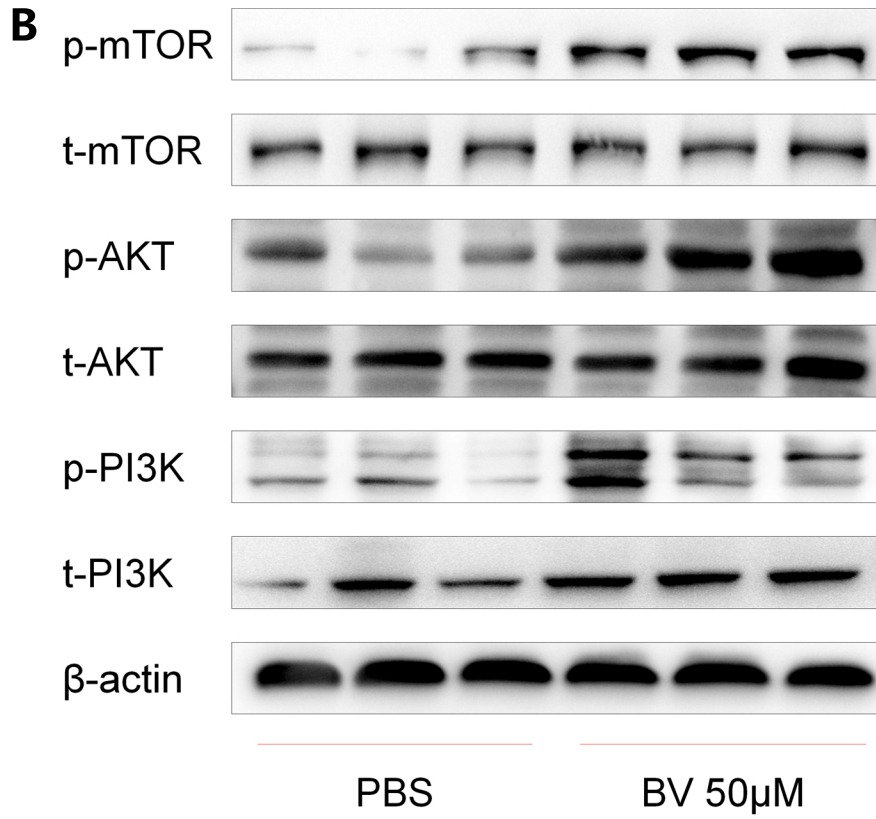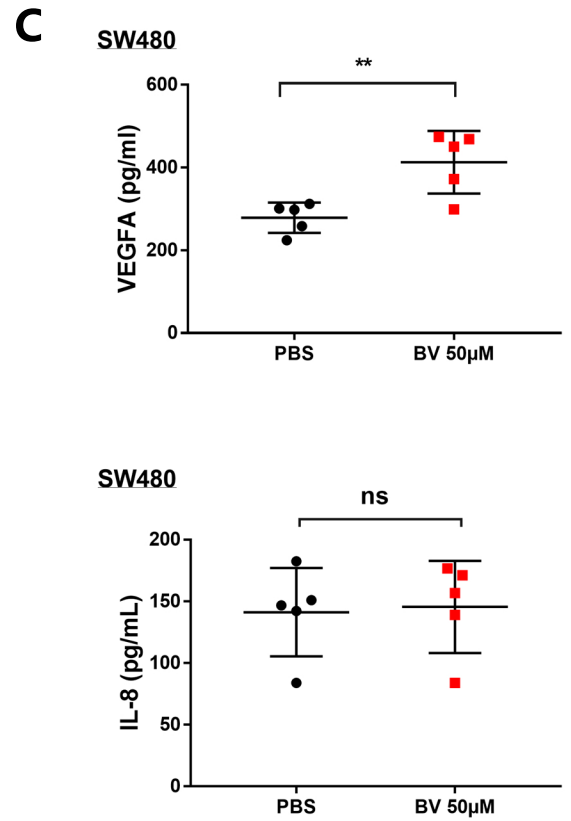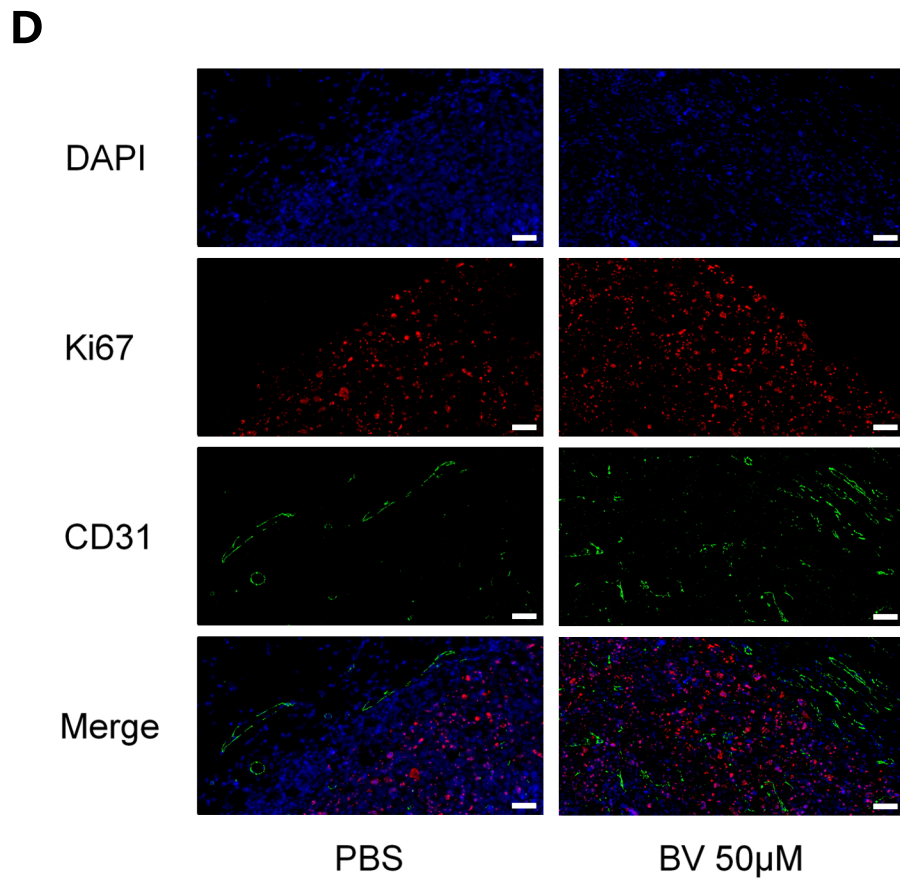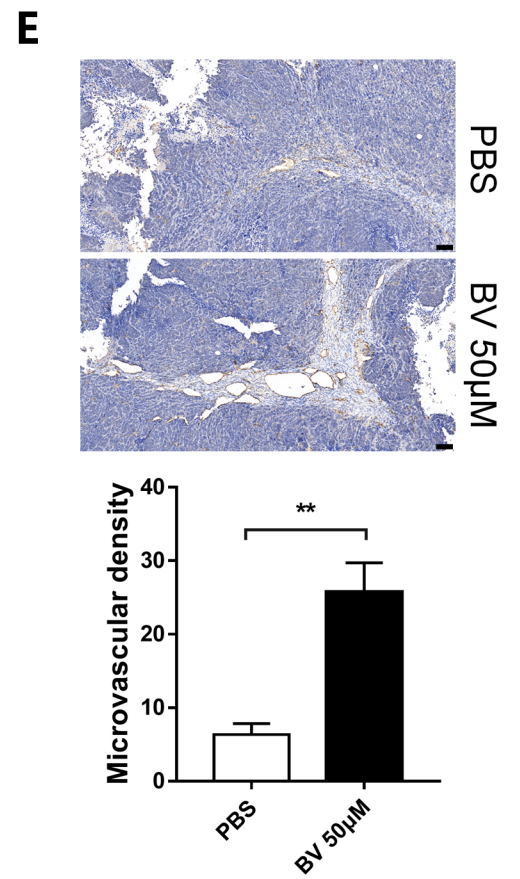

Supplement: Supplementary file 3 — Additional file 3: BV promotes proliferation and angiogenesis in xenograft models of SW480. (A) Representative images of subcutaneous tumors in nude mice injected SW480 cells treated with or without BV. Both the volume and weight of subcutaneous tumor were shown in the right panel. (B) PI3K/AKT/mTOR pathway was detected in mice tumor by western blot analysis. (C) The concentration of IL-8 and VEGFA in mice tumors were detected by ELISA. (D) The expression of Ki67 and CD31 in subcutaneous tumors of nude mice were assessed by immunofluorescence staining. Representative images were shown. Scale bar represents 50μm. (E) IHC analysis demonstrated the expression of CD31 in subcutaneous tumors of nude mice. Bars of the right panel represent the microvascular density. Scale bar represents 100μm. **, P < 0.01. [file 12967_2023_3929_MOESM3_ESM.pdf]
