# Supplementary material for: Enterococcus faecalis promotes the progression of colorectal cancer via its metabolite: biliverdin
Source: J Transl Med. 2023 Feb 2;21:72. doi: 10.1186/s12967-023-03929-7 (PMC9896694; doi:10.1186/s12967-023-03929-7)
Supplement: Supplementary file 4 — Additional file 4: Metabolomics analysis of Efa with two different MOI (100:1 and 1:1). A: PCA score plot of metabolomics. The orange dots represent Efa in MOI 100:1, and the blue dots represent Efa in MOI 1:1. B: KEGG analysis for the various differentially expressed signal pathway. The dots represented various pathways. Pathways impact was represented by the area of each dots. The p-value was represented by a color scale from blue (relatively lower significance) to orange (relatively higher significance). C: Heatmap of differentially metabolites. The p-values were represented by a color scale from blue (relatively lower expression) to red (relatively higher expression). Each column represented individual sample, and each row represented a single metabolite. The left panel is Efa with MOI 1:1, and the right part is Efa with MOI 100:1. [file 12967_2023_3929_MOESM4_ESM.pdf]

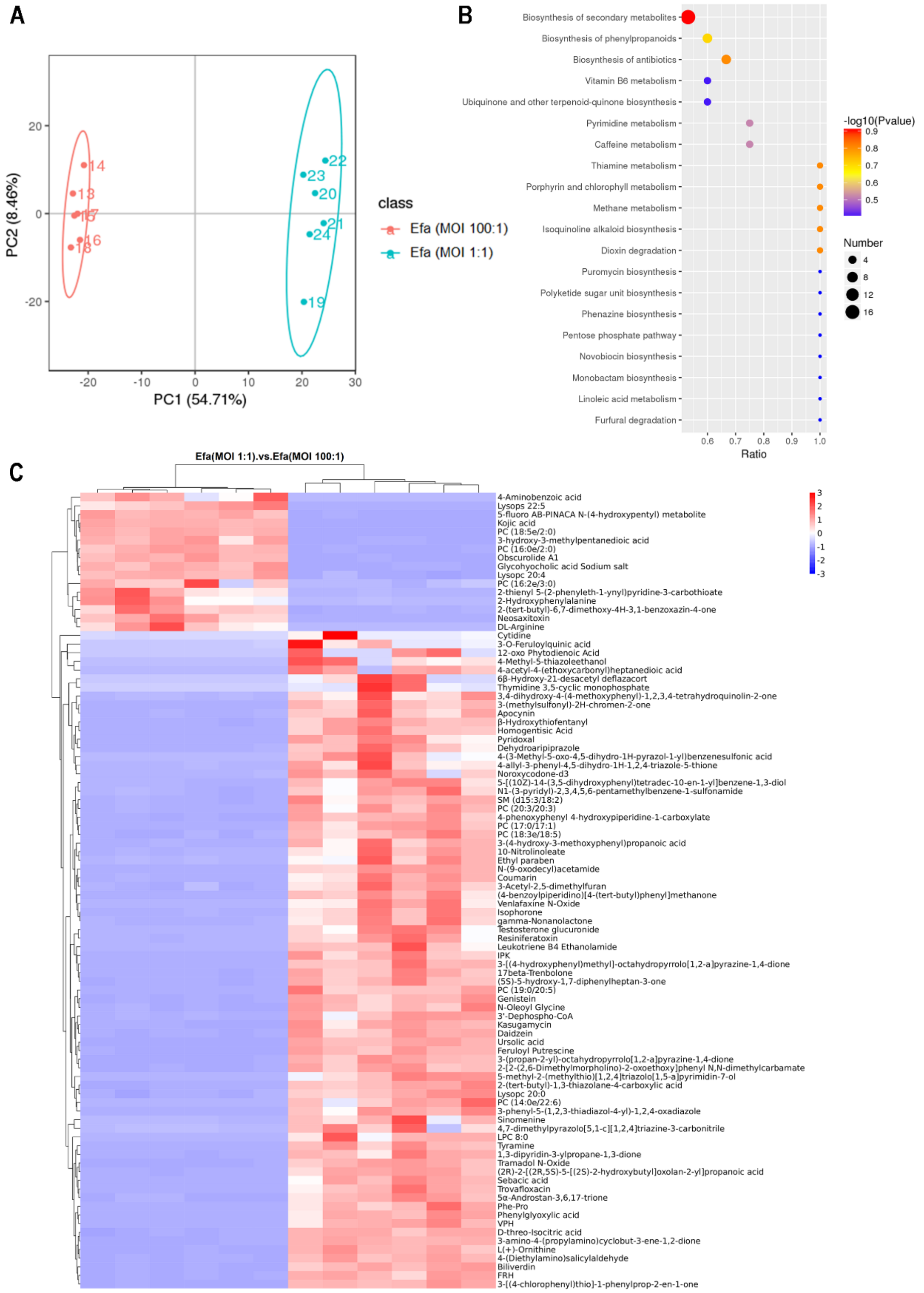

Additional file 4: Metabolomics analysis of Efa with two different MOI (100:1 and 1:1).

A: PCA score plot of metabolomics. The orange dots represent Efa in MOI 100:1, and the blue dots represent Efa in MOI 1:1. B: KEGG analysis for the various differentially expressed signal pathway. The dots represented various pathways. Pathways impact was represented by the area of each dots. The p-value was represented by a color scale from blue (relatively lower significance) to orange (relatively higher significance). C: Heatmap of differentially metabolites. The p-values were

represented by a color scale from blue (relatively lower expression) to red (relatively higher expression). Each column represented individual sample, and each row represented a single metabolite. The left panel is Efa with MOI 1:1, and the right part is Efa with MOI 100:1.
