# Supplementary material for: Enterococcus faecalis promotes the progression of colorectal cancer via its metabolite: biliverdin
Source: J Transl Med. 2023 Feb 2;21:72. doi: 10.1186/s12967-023-03929-7 (PMC9896694; doi:10.1186/s12967-023-03929-7)
Supplement: Supplementary file 5 — Additional file 5: Table S1. The relative quantitative differences of BV in Efa with two different MOI (100:1 and 1:1). [file 12967_2023_3929_MOESM5_ESM.docx]

**Table S1.** The relative quantitative differences of BV in Efa with two different MOI (100:1 and 1:1).

| Group | | BV (Relative Quantification) |
| --- | --- | --- |
| Efa (MOI 100:1) | Sample 1 | 69218791.86 |
|  | Sample 2 | 58035623.1 |
|  | Sample 3 | 69314307.31 |
|  | Sample 4 | 73074903.59 |
|  | Sample 5 | 52765305.9 |
|  | Sample 6 | 60637500.78 |
| Efa (MOI 1:1) | Sample 1 | 3063048.487 |
|  | Sample 2 | 3000769.848 |
|  | Sample 3 | 1977831.344 |
|  | Sample 4 | 2223554.772 |
|  | Sample 5 | 2340758.533 |
|  | Sample 6 | 1578806.304 |
| Efa (MOI 100:1)  vs  Efa (MOI 1:1) | P value | 8.02E-09 |
|  | FC | 27.00406505 |
|  | Log2FC | 4.755105 |
|  | VIP | 1.378387671 |

BV, biliverdin; Efa, *Enterococcus faecalis*; MOI, multiplicity of infection; FC, fold change; VIP, variable important in projection.
